# Neonatal 2′-Fucosyllactose Supplementation Modulates Exploratory Behavior and Hippocampal Synaptic Plasticity in Rats Through Gut Microbiota and Metabolic Remodeling

**DOI:** 10.3390/foods15173151

**Published:** 2026-09-05

**Authors:** Miao Yu, Jin Wang, Ruixin Kou, Bingye Xu, Weiqian Zhang, Ying Zhang, Jingmin Liu, Shuo Wang

**Affiliations:** 1 Tianjin Key Laboratory of Food Science and Health, School of Medicine, Nankai University, Tianjin 300071, China; yumiao222@mail.nankai.edu.cn (M.Y.);; 2 Research Institute of Public Health, Nankai University, Tianjin 300071, China

**Keywords:** behavioral development, gut microbiota, gut–brain axis, 2′-fucosyllactose, neonatal nutrition

## Abstract

2′-Fucosyllactose (2′-FL) represents a significant constituent within human milk oligosaccharides, and its benefits for intestinal health and neurodevelopment in infants and young children have been widely recognized. However, the mechanism by which 2′-FL influences early-life behavioral development through the gut–brain axis remains incompletely understood. In this study, we investigated the effects of neonatal 2′-FL supplementation on behavioral development in Sprague-Dawley (SD) rats and explored its potential microbiota-metabolite regulatory mechanisms. After 4 consecutive weeks of oral gavage beginning on postnatal day 2, rats receiving 2′-FL showed significantly increased average movement speed and central-area exploration time in the open field test. From a systemic perspective, 2′-FL lowered blood diamine oxidase (DAO) and D-lactic acid (D-LA) concentrations while elevating colonic mRNA levels of tight junction protein-related genes, consequently enhancing intestinal barrier stability. In addition, 16S rRNA sequencing showed that 2′-FL reshaped the gut microbiota structure and significantly enriched *Lactobacillus*, *Bacteroides*, and short-chain fatty acid (SCFA)-associated taxa represented by Lachnospiraceae_NK4A136_group. Untargeted metabolomics revealed that 2′-FL induced systemic metabolic remodeling, characterized by upregulation of the bioactive metabolites dehydroepiandrosterone sulfate (DHEA-S) and deoxycholic acid (DCA), and downregulation of the glucocorticoid-related corticosterone. Western blot validation further showed higher hippocampal protein expression of postsynaptic density protein 95 (PSD95), brain-derived neurotrophic factor (BDNF), and synaptophysin in the 2′-FL group, supporting enhanced synaptic plasticity-related signaling. In summary, neonatal 2′-FL intervention enhanced exploratory behavior and was accompanied by increased hippocampal synaptic plasticity-related protein expression in rats, potentially through coordinated regulation of gut microbiota composition and systemic metabolism.

## 1. Introduction

Nutritional intervention in early life has profound and lasting effects on individual health, especially on neurological development [1]. Breastfeeding is considered to be the best food for babies and young children, not only because of its balanced macronutrient composition but also because it is rich in a variety of bioactive components. Among these, human milk oligosaccharides (HMOs) represent the third major solid constituent following lactose and lipids [2,3]. HMOs cannot be directly digested and absorbed by the host; instead, they exert biological effects by shaping the gut microbiota, maintaining mucosal barrier integrity, and regulating immune responses [4,5]. 2′-FL is the most abundant and representative HMO, accounting for approximately 31% of the total HMO content, and its concentration is particularly high in colostrum [6,7].

The prebiotic properties of 2′-FL have been widely documented. It can selectively promote the growth of beneficial gut microbes, thereby optimizing intestinal microbial structure [8,9,10]. In addition, 2′-FL can enhance intestinal epithelial barrier function by upregulating tight junction-related molecules, competitively inhibiting the adhesion of pathogenic bacteria, and regulating local mucosal immunity, all of which are crucial for maintaining intestinal homeostasis [11,12]. Over the past few years, the concept of the gut–brain axis has attracted increasing attention. The intestinal tract interacts reciprocally with the brain via neural, endocrine, immune, and metabolic routes. This complex network influences neural development, synapse formation, myelination, and subsequent behavioral phenotypes [13,14]. Infancy functions as a vital period when intestinal microbial settlement and nervous system maturation intersect significantly. Nutritional intervention at this stage may therefore exert long-term imprinting effects on microbiota-brain interactions.

In recent years, the potential of 2′-FL to regulate central nervous system development has gradually attracted attention. Clinical and preclinical studies have shown that HMOs can affect brain structure and function. In rodent models, they can enhance long-term potentiation (LTP) and improve learning- and memory-related performance [15,16]. However, current studies still have several limitations: most have used adult animal models, direct intracerebral administration, or focused mainly on behavioral phenotypes without clarifying the systemic molecular mechanisms connecting the gut and brain. Specifically, the mechanism by which neonatal gut microbiota changes induced by 2′-FL are translated into circulating metabolic signals and associated with hippocampal synaptic plasticity has not been fully clarified. The regulatory pathway of 2′-FL during the critical period of early neurodevelopment therefore still requires systematic experimental verification.

To address this gap, we aimed to determine whether neonatal 2′-FL supplementation links gut microbiota remodeling with circulating metabolic changes, intestinal barrier integrity, and hippocampal synaptic plasticity-related molecular profiles, including postsynaptic density protein 95 (PSD95), brain-derived neurotrophic factor (BDNF), and synaptophysin, thereby influencing early-life behavioral performance. To test this hypothesis, we used neonatal SD rats as a model and adopted a multidimensional research strategy integrating behavioral analysis, intestinal mucosal barrier assessment, 16S rRNA microbiota sequencing, untargeted serum metabolomics, immunofluorescence staining, and Western blot detection. By analyzing the gut microbiota-metabolite-brain axis, this research offers empirical evidence and mechanistic insight into the possible utilization of 2′-FL in enhancing neural maturation within infant feeding.

## 2. Materials and Methods

### 2.1. Animal Grouping and Intervention Program

All animal experimental protocols were approved by the Laboratory Animal Management and Utilization Committee of Nankai University (Approval No.: 2025-SYDWLL-000665) and were conducted in accordance with national guidelines for the welfare of laboratory animals. Two pregnant Sprague-Dawley (SD) rats were obtained from Beijing Vital River Laboratory Animal Technology Co., Ltd. (Beijing, China) and housed in the laboratory animal facility under controlled environmental conditions, with a temperature of 22 ± 2 °C relative humidity of 50 ± 15%, and a 12 h light/12 h dark cycle. Animals had free access to standard chow and water. Bedding suitable for pregnant dams and neonatal pups was provided as environmental enrichment.

After birth, neonatal rats were allocated into two sex-balanced groups, with 8 pups in each group: the control group (CON; 4 males and 4 females) and the 2′-FL intervention group (2′-FL; 4 males and 4 females). To minimize litter effects, pups from each litter were allocated to both groups whenever possible. Group allocation was performed by balancing sex and litter origin. The individual pup was considered the experimental unit. Pups were housed with their respective dams during lactation and were weaned on postnatal day 21. Body weight was recorded daily throughout the intervention period. The sample size was determined based on previous related neonatal animal studies involving 2′-FL intervention [17]. From postnatal day 2, pups in the 2′-FL group received 1 g/kg 2′-FL (purity ≥ 95%, dissolved in sterile PBS) by oral gavage once daily, whereas pups in the CON group received an equal volume of sterile PBS. The intervention lasted for 4 consecutive weeks. Animals were monitored daily for general appearance, activity, feeding status, body weight changes, and signs of distress.

Before tissue collection, behavioral testing was performed using the open field test. The primary behavioral outcomes were average movement speed and central-area residence time. At the end of the intervention, animals were fasted for 12 h and deeply anesthetized with ether according to the approved protocol. Blood was collected from the retro-orbital venous plexus, followed by euthanasia by cervical dislocation. Blood samples were collected in non-anticoagulant tubes and centrifuged to obtain serum for ELISA and metabolomics analyses. Serum, brain, colon, fecal, and other samples were collected for subsequent analyses.

### 2.2. Open Field Test

Prior to sacrifice at the conclusion of the intervention, an open field device was utilized to assess locomotor activity and exploratory/anxiety-related actions in rats. Every animal was positioned at the center of the arena, and its movement path was captured for 10 min via the Any-maze video monitoring tool [18]. The behavioral metrics evaluated comprised mean movement velocity and dwelling time within the central zone (25% of the overall space).

### 2.3. Assessment of Intestinal Barrier Integrity and Mucosal Immune-Related Markers

Serum levels of D-lactic acid (D-LA), lipopolysaccharide/endotoxin (LPS), diamine oxidase (DAO), and secretory immunoglobulin A (s-IgA) were measured using commercial enzyme-linked immunosorbent assay (ELISA) kits supplied by Jiangsu Enzyme-Labeling Biotechnology Co., Ltd. (Yanchen, China), according to the manufacturer’s protocols. The catalog numbers were as follows: rat D-LA ELISA kit (MB-7288B); rat LPS/endotoxin ELISA kit (MB-7290B); rat DAO ELISA kit (MB-6915B); and rat s-IgA ELISA kit (MB-1970B). Standard curve regression equations were calculated using ELISACalc software (V0.1) based on absorbance values measured at 450 nm (OD450) and the corresponding standard concentrations. A four-parameter logistic curve was used as the fitting model, and the levels of each index were calculated accordingly.

### 2.4. Real-Time Quantitative PCR Analysis of Colonic Tight Junction-Related Gene Expression

TRIzol reagent was used to extract total RNA from colon tissue. After determination of RNA concentration and purity using a spectrophotometer, reverse transcription was performed to synthesize cDNA [19]. Primers derived from reverse-transcribed cDNA served as templates for real-time quantitative PCR utilizing Vazyme^®^ ChamQ Universal SYBR qPCR Master Mix. β-Actin functioned as the internal standard for normalization, with the relative mRNA levels of different groups determined relative to the CON group as the reference. Quantification of target gene expression was performed via the 2^−ΔΔCt^ approach. Primers are listed in Supplementary Table S1.

### 2.5. 16S rRNA Gene Sequencing

Fresh fecal samples were collected before euthanasia after the 12 h fasting period, immediately frozen, and stored at −80 °C until DNA extraction. Microbial genomic DNA was extracted from fecal samples collected from rats in different groups. The V3-V4 region of the bacterial 16S rRNA gene was amplified using primers 338F (5′-ACTCCTACGGGAGGCAGCA-3′) and 806R (5′-GGACTACHVGGGTWTCTAAT-3′). Amplicons were sequenced using the Illumina NovaSeq platform (San Diego, CA, USA). Raw reads were quality-filtered, denoised, and processed using QIIME 2 (v2020.2) to generate feature tables and taxonomic annotations. Alpha diversity, beta diversity-based principal component analysis (PCA), and linear discriminant analysis effect size (LEfSe) analysis with linear discriminant analysis (LDA) were then performed. Spearman correlation analysis was used to evaluate associations between key bacterial taxa and behavioral, intestinal barrier, metabolic, and hippocampal synaptic plasticity-related indicators.

### 2.6. Untargeted Serum Metabolomics Analysis

Untargeted serum metabolomics was performed using ultra-high-performance liquid chromatography-tandem mass spectrometry (UHPLC-MS/MS). Serum samples (100 µL) were placed in Eppendorf tubes and mixed with prechilled 80% methanol. After vortexing, samples were incubated on ice for 5 min and centrifuged at 15,000× *g* and 4 °C for 20 min. Part of the supernatant was diluted with LC-MS-grade water to a final methanol concentration of 53%, transferred to a fresh tube, and centrifuged again at 15,000× *g* and 4 °C for 20 min. The final supernatant was injected into the LC-MS/MS system. UHPLC-MS/MS analyses were performed using a Vanquish UHPLC system (Thermo Fisher, Scientific, Germering, Germany) coupled with an Orbitrap Q Exactive HF or Orbitrap Q Exactive HF-X mass spectrometer (Thermo Fisher, Germany). Samples were separated on a Hypersil Gold column (100 × 2.1 mm, 1.9 µm) at a flow rate of 0.2 mL/min. The mobile phases were 0.1% formic acid in water (eluent A) and methanol (eluent B). The gradient was set as follows: 2% B for 1.5 min, 2–85% B for 3 min, 85–100% B for 10 min, 100–2% B for 10.1 min, and 2% B for 12 min. The mass spectrometer was operated in positive/negative ion mode with a spray voltage of 3.5 kV, capillary temperature of 320 °C, sheath gas flow rate of 35 psi, auxiliary gas flow rate of 10 L/min, S-lens RF level of 60, and auxiliary gas heater temperature of 350 °C. Raw data were processed using Compound Discoverer 3.3 (Thermo Fisher), and metabolite annotation was performed using mzCloud, mzVault (2.3), MassList, KEGG (Release 119.0), HMDB (5.0), and LIPIDMaps databases. PCA and partial least squares-discriminant analysis (PLS-DA) were applied to evaluate differences in metabolic profiles between groups. Differential metabolites in this study were screened using the criteria variable importance in projection (VIP) > 1, *p* < 0.05, and fold change (FC) > 1.5 or <0.67, and pathway enrichment analysis was conducted using the KEGG database.

### 2.7. Quantitative Analysis of PSD95 Immunofluorescence in the Hippocampus

Paraffin-embedded hippocampal sections were prepared through graded ethanol dehydration, xylene clearing, paraffin infiltration, embedding, sectioning, and slide mounting. Briefly, hippocampal tissues were dehydrated in 75%, 85%, 95%, 100%, and 100% ethanol for 1 h at each step, cleared in two xylene baths for 20 min and 30 min, respectively, and infiltrated in three paraffin baths for 1 h, 1.5 h, and 2 h, respectively. The wax blocks were trimmed after cooling, and 4-µm sections were cut using a paraffin microtome. Sections were floated in a water bath at approximately 40 °C, mounted onto glass slides, air-dried, baked at 60 °C for 1 h, and further dried in an oven for 1 h. For immunofluorescence staining, sections were deparaffinized, rehydrated, subjected to heat-mediated antigen retrieval, and blocked using PBS mixed with 0.3% Triton X-100 and 5% bovine serum albumin, followed by overnight incubation at 4 °C with anti-PSD95 monoclonal antibody (1:200, Abcam, Cambridge, UK). These sections were subsequently incubated for 1 h at room temperature in the dark with Alexa Fluor 594-conjugated goat anti-mouse IgG secondary antibody (1:500, Invitrogen, Carlsbad, CA, USA), while nuclei were stained with DAPI [20]. Fluorescent images were observed using an OLYMPUS CKX43-LP microscope (Olympus, Tokyo, Japan), and whole-slide images were acquired using a Pannoramic 250 FLASH slide scanner (3DHISTECH, Budapest, Hungary). ImageJ software (1.54r) was used to measure the mean fluorescence intensity and density of PSD95-positive puncta within the hippocampal CA1 area.

### 2.8. Western Blot Analysis of Hippocampal Synaptic Plasticity-Related Proteins

Total hippocampal proteins were extracted using a whole-protein extraction kit (wanleibio, Shenyang, China, WLA019) and quantified using a bicinchoninic acid (BCA) protein assay kit (wanleibio, WLA004), according to the manufacturer’s instructions. Equal amounts of protein were separated by sodium dodecyl sulfate-polyacrylamide gel electrophoresis (SDS-PAGE) using an SDS-PAGE gel rapid preparation kit (wanleibio, WLA013) and transferred onto polyvinylidene fluoride (PVDF) membranes (Millipore, Burlington, MA, USA, IPVH00010). After blocking, membranes were incubated overnight at 4 °C with primary antibodies against PSD95 (1:1000, wanleibio, WL05046), BDNF (1:1000, wanleibio, WL0168), synaptophysin (1:1000, wanleibio, WL03058), and beta-actin as the internal control. Membranes were then incubated with HRP-conjugated secondary antibody (1:5000, 37 °C, 45 min). Protein bands were visualized by chemiluminescence using ECL reagent (wanleibio, WLA006), and grayscale values were quantified using ImageJ (1.54r). Relative protein expression was calculated after normalization to beta-actin.

### 2.9. Statistical Analysis

Analytical procedures for the experimental data were performed using GraphPad Prism 10.0.1. Every outcome is expressed as mean ± standard deviation (SD). Data that followed a Gaussian distribution were evaluated using the independent-samples *t* test, whereas data deviating from a Gaussian distribution were assessed using the Mann–Whitney U test. Statistical significance was established as *p* < 0.05. The levels of significance are denoted as * *p* < 0.05, ** *p* < 0.01, and *** *p* < 0.001.

## 3. Results

### 3.1. Effect of Neonatal 2′-FL Intervention on Body Weight and Organ Indices in Rats

**Figure 1:**
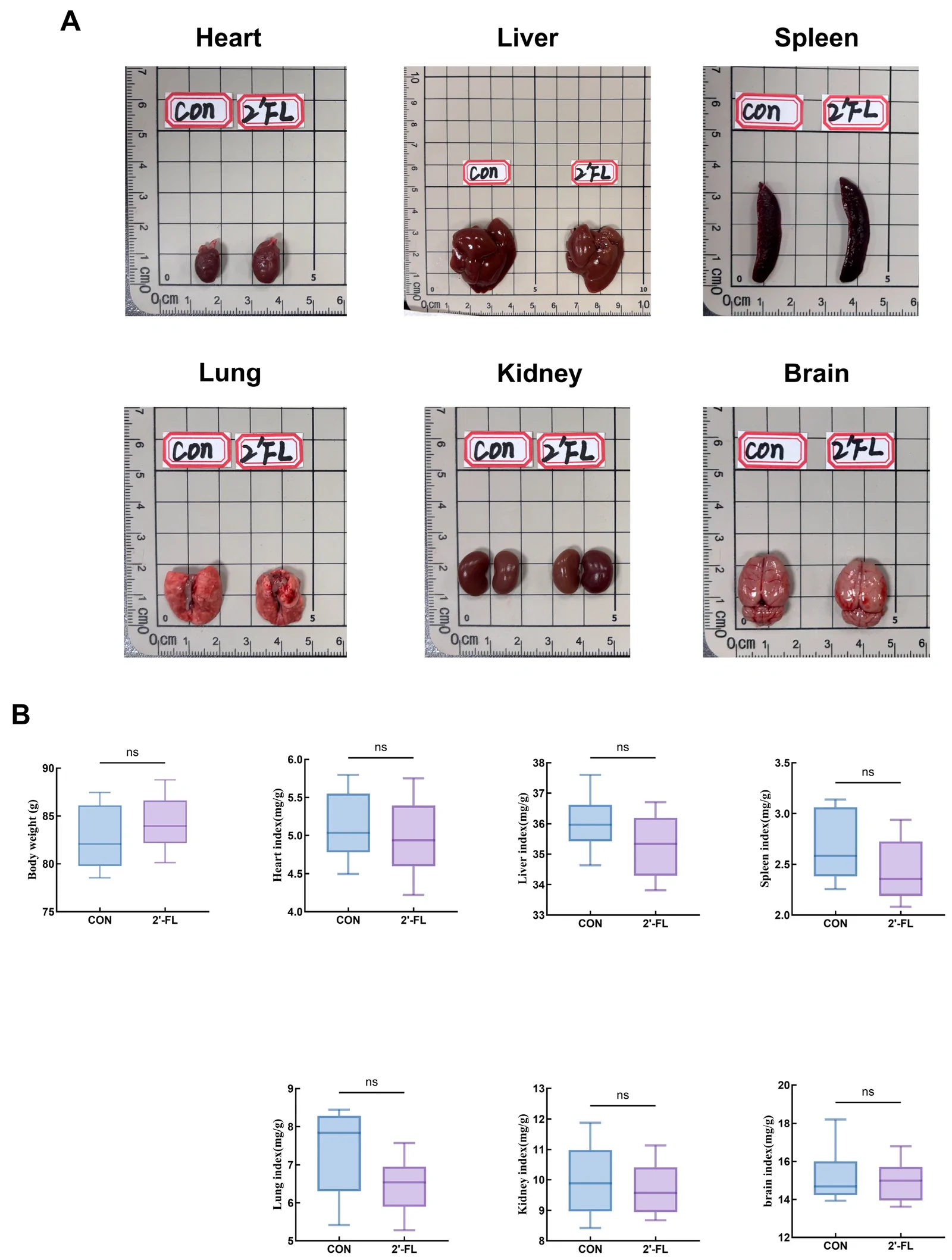
Body weight, representative organ images, and organ indices of neonatal rats after early-life 2′-FL intervention. (**A**) Representative images of major organs, including brain, kidney, lung, heart, spleen, and liver. (**B**) Body weight before sacrifice and organ indices of the brain, kidney, lung, heart, spleen, and liver. Data are presented as mean ± SD. Statistical analysis was performed using an independent-samples *t* test or Mann–Whitney U test, as appropriate.ns, not significant.

To evaluate whether early-life 2′-FL supplementation affected general growth and basic physiological status, body weight before sacrifice and organ indices of the heart, liver, spleen, lung, kidney, and brain were analyzed. Representative organ images are shown in Figure 1A, and body weight and organ index data are shown in Figure 1B. Compared with the CON group, no statistically significant differences were observed in body weight or the major organ indices of rats in the 2′-FL group (*p* > 0.05) (Figure 1B). These findings indicate that neonatal 2′-FL supplementation at 1 g/kg did not cause obvious alterations in overall growth or major organ relative weights, providing a basic safety background for subsequent behavioral, metabolic, microbiota, and hippocampal analyses.

### 3.2. 2′-FL Significantly Enhances Locomotor Activity and Exploratory Behavior in Rats Receiving Neonatal Supplementation

**Figure 2:**
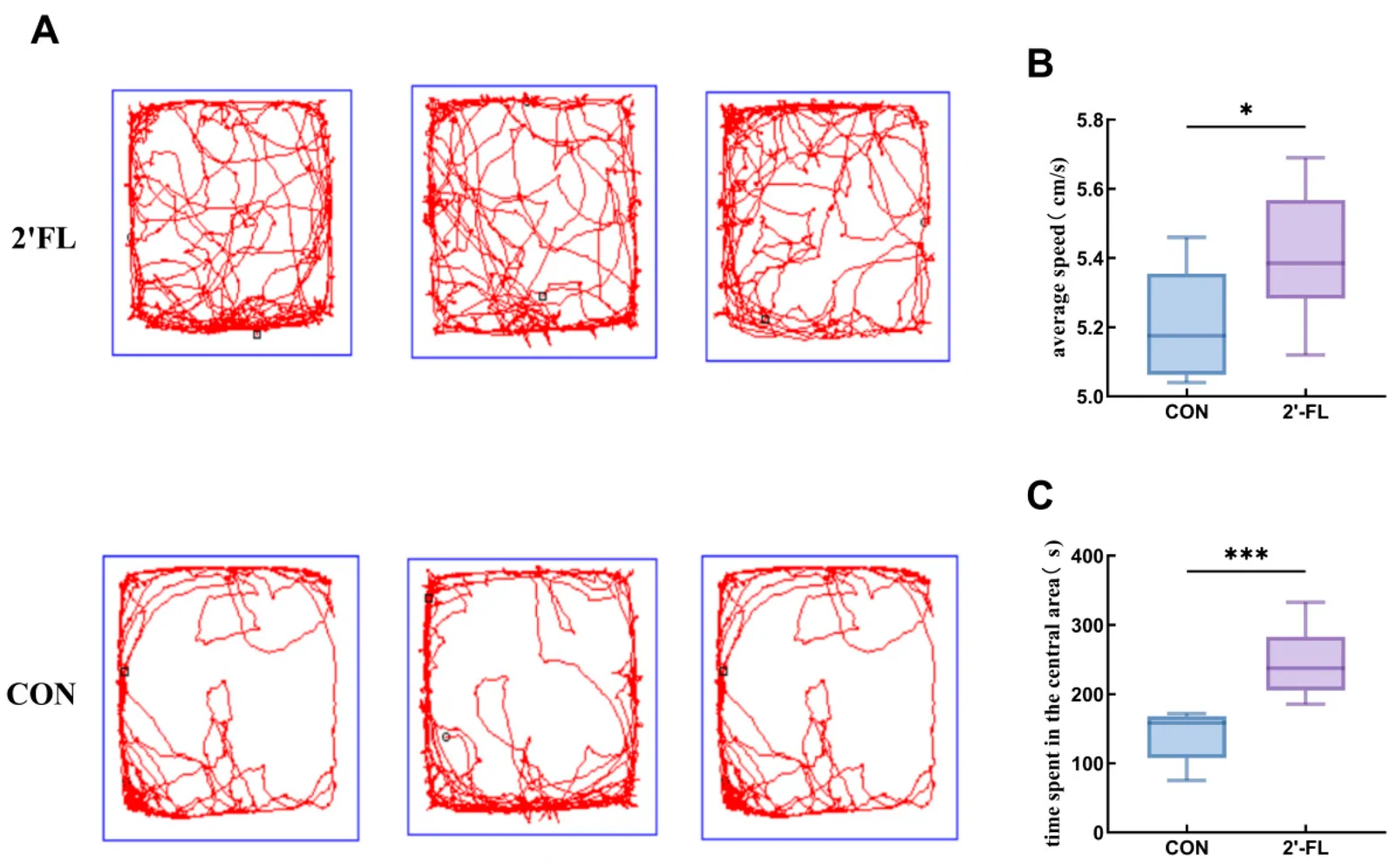
Exploratory behavior and locomotor activity of neonatal rats. (**A**) Representative movement trajectories; (**B**) average speed during the 10-min open field test; (**C**) proportion of residence time in the central area. Data are presented as mean ± SD. Statistical analysis was performed using an independent-samples *t* test or Mann–Whitney U test, as appropriate.* *p* < 0.05, and *** *p* < 0.001.

Locomotor activity and exploratory/anxiety-like behavior were evaluated by the open field test. Movement tracks for both experimental cohorts are depicted in Figure 2A. Mean locomotive velocity in the 2′-FL cohort exhibited a notably greater magnitude compared to the CON cohort (*p* < 0.05) (Figure 2B). Simultaneously, the relative duration spent within the field’s central sector was markedly elevated (*p* < 0.05) (Figure 2C). This behavioral pattern is generally interpreted as enhanced exploratory activity and reduced anxiety-like behavior in a novel environment. The concurrent increase in average speed and central exploration time suggests that neonatal 2′-FL intervention may positively influence early-life behavioral performance, potentially through modulation of nervous system excitability or energy metabolism [21].

### 3.3. 2′-FL Improves Intestinal Permeability and Enhances Intestinal Barrier Function

**Figure 3:**
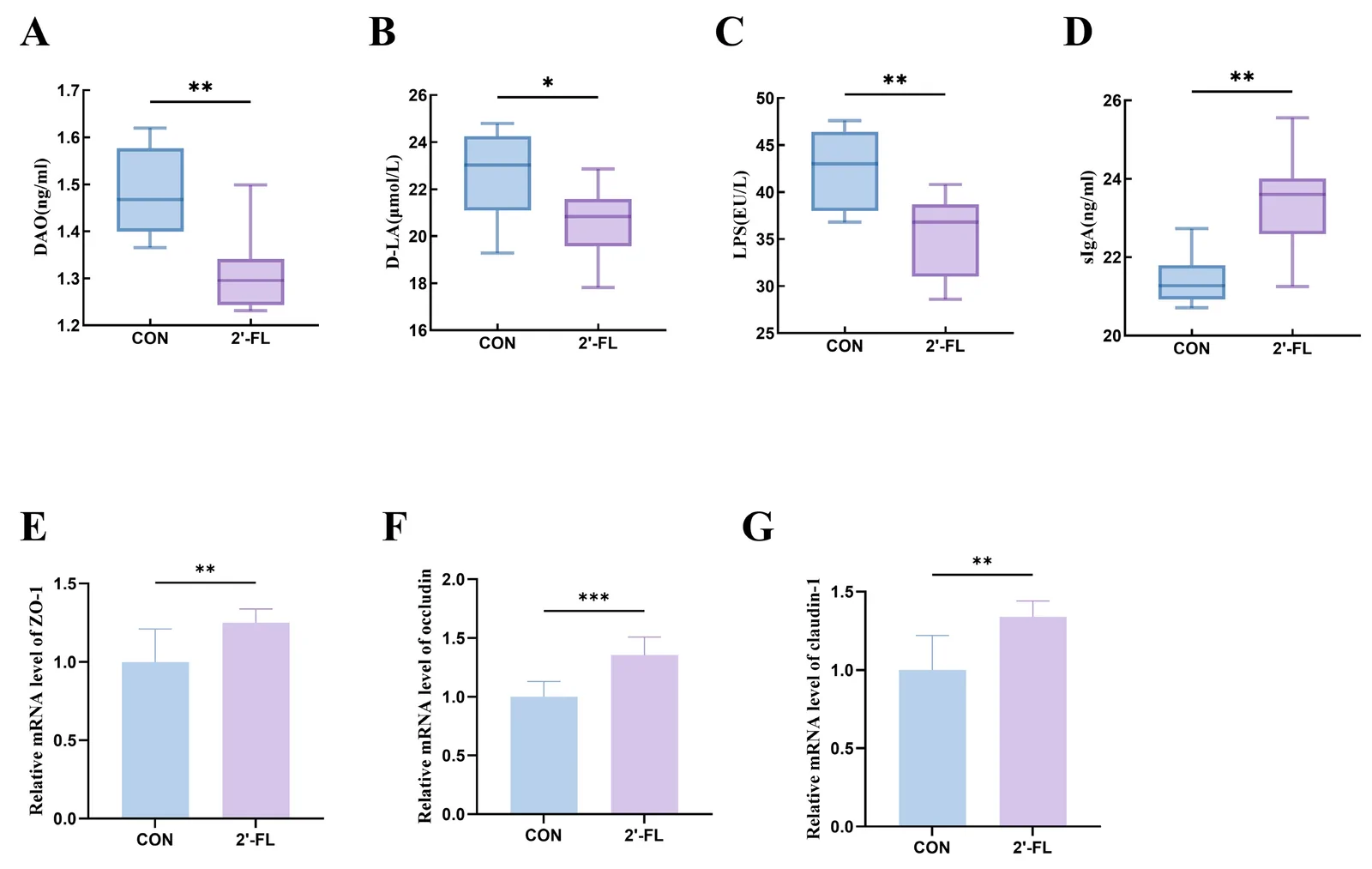
Intestinal barrier function and tight junction protein expression. (**A**–**D**) ELISA detection of diamine oxidase (DAO) (**A**), D-lactic acid (D-LA) (**B**), lipopolysaccharide/endotoxin (LPS) (**C**), and secretory immunoglobulin A (s-IgA) (**D**); (**E**–**G**) RT-qPCR detection of relative mRNA expression of zonula occludens-1 (ZO-1) (**E**), occludin (**F**), and claudin-1 (**G**) in colon tissue. Data are presented as mean ± SD. Statistical analysis was performed using an independent-samples *t* test or Mann–Whitney U test, as appropriate.* *p* < 0.05, ** *p* < 0.01, and *** *p* < 0.001.

To investigate the correlation between behavioral outcomes and intestinal epithelial integrity, intestinal permeability- and barrier-related indicators were analyzed. Relative to the CON group, serum concentrations of the intestinal epithelial damage markers diamine oxidase (DAO) and D-lactic acid (D-LA) were notably decreased in the 2′-FL group (Figure 3A,B). The serum level of the bacterial translocation-related marker lipopolysaccharide/endotoxin (LPS) was likewise markedly diminished (Figure 3C), while the concentration of secretory immunoglobulin A (s-IgA) was substantially elevated (Figure 3D). Furthermore, following 2′-FL administration, the colonic mRNA expression levels of the vital tight junction-related genes zonula occludens-1 (ZO-1), occludin, and claudin-1 were significantly upregulated (all *p* < 0.01) (Figure 3E–G). These findings demonstrate that neonatal 2′-FL supplementation can effectively reinforce intestinal mucosal barrier integrity.

### 3.4. 2′-FL Reshapes the Gut Microbiota Structure by Enriching Beneficial Taxa

**Figure 4:**
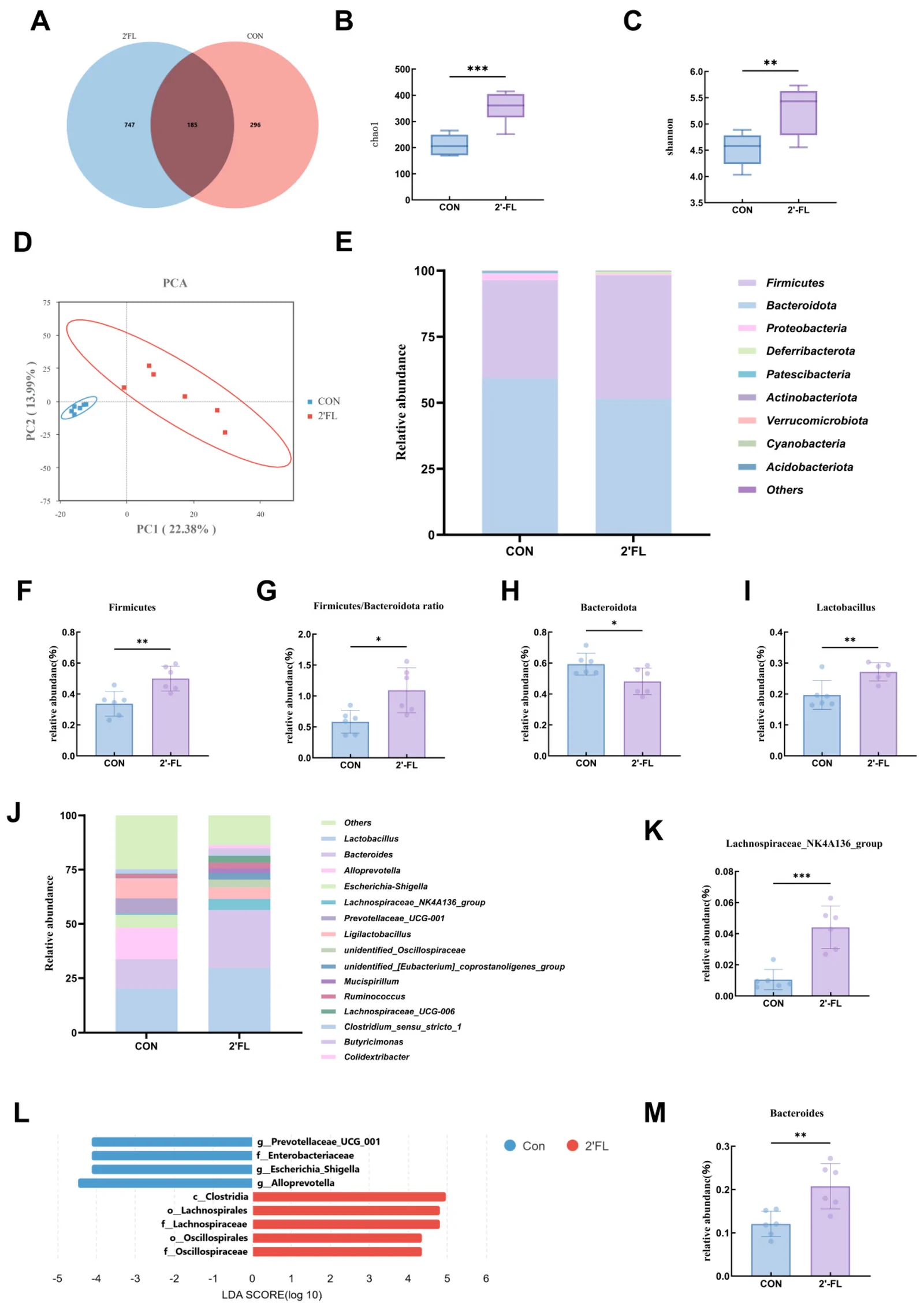
Gut microbiota composition and microbial ecosystem remodeling. (**A**) Venn diagram; (**B**) Chao1 index; (**C**) Shannon index; (**D**) PCA plot; (**E**,**J**) microbiota composition at the phylum level (**E**) and genus level (**J**); (**F**–**H**) relative abundances of the dominant phyla; (**I**,**K**,**M**) relative abundances of key bacterial taxa; (**L**) LEfSe analysis (LDA score > 4.0). Dots represent individual biological replicates.Data are presented as mean ± SD for quantitative panels. Statistical analysis was performed using an independent-samples *t* test or Mann–Whitney U test, as appropriate.* *p* < 0.05, ** *p* < 0.01, and *** *p* < 0.001.

To explore the impact of 2′-FL on the intestinal microbiome, 16S rRNA sequencing was conducted on fecal samples from rats across various cohorts. The Venn diagram highlighted the common and specific microbial profiles within the gut of both cohorts (Figure 4A). The Chao1 index was used to estimate microbial richness, whereas the Shannon index was used to evaluate both microbial richness and evenness. Relative to the CON group, the 2′-FL treatment notably elevated both the Chao1 and Shannon values (Figure 4B,C), demonstrating that 2′-FL boosted the variety and complexity of intestinal microbial populations in neonatal rats. Principal component analysis (PCA) derived from beta diversity demonstrated distinct clustering between these two groups, suggesting that 2′-FL triggered significant alterations in the gut microbiota composition (Figure 4D).

Phylum-level taxonomic characterization indicated that intestinal microbial communities were mainly dominated by Firmicutes and Bacteroidota (Figure 4E). Notably, 2′-FL intervention altered the relative abundance of these dominant phyla, indicating a clear compositional shift in the gut microbial community (Figure 4F–H). This result suggests that 2′-FL acts as a prebiotic substrate capable of remodeling the core gut microbiota.

At the genus level, specific key taxa were further visualized to characterize this compositional restructuring (Figure 4J). The results showed that 2′-FL markedly altered the intestinal microbial profile. Specifically, the relative abundances of *Lactobacillus*, a genus widely associated with beneficial host effects, and Lachnospiraceae_NK4A136_group, an SCFA-associated taxon, were substantially enriched (Figure 4I,M). Simultaneously, the proportion of *Bacteroides* showed a notable increase, potentially linked to enhanced intestinal epithelial integrity (Figure 4K).

Subsequent linear discriminant analysis effect size (LEfSe) analysis (linear discriminant analysis [LDA] score > 4.0) was used to identify differentially abundant taxonomic biomarkers. The CON microbiome was predominantly characterized by enrichment of taxa such as genus *Escherichia-Shigella*, family Enterobacteriaceae, and genus *Alloprevotella*. In contrast, the 2′-FL group exhibited robust enrichment of potentially beneficial and SCFA-associated taxa, primarily species of *Lactobacillus intestinalis*, together with lineages including the family Lachnospiraceae and the order Lachnospirales (Figure 4L). These compositional changes are consistent with the observed improvements in mucosal barrier function [22,23].

In summary, 2′-FL intervention reshaped the gut microbiota structure in rats, with the core change being significant enrichment of taxa associated with probiotic functions and SCFA production. Such results indicate that 2′-FL might provide physiological advantages to the body by functioning as a prebiotic and enhancing gut microbial balance.

### 3.5. Effects of 2′-FL on Serum Metabolites in Rats

Untargeted metabolic profiling of rodent serum was conducted to explore the prospective systemic metabolic shifts linked to 2′-FL supplementation. Following multivariate statistical evaluation (VIP > 1, *p* < 0.05) and fold-change filtering (FC > 1.5 or <0.67), multiple substantially modified metabolites were detected, subsequently followed by pathway enrichment analysis of the related biological processes.

**Figure 5:**
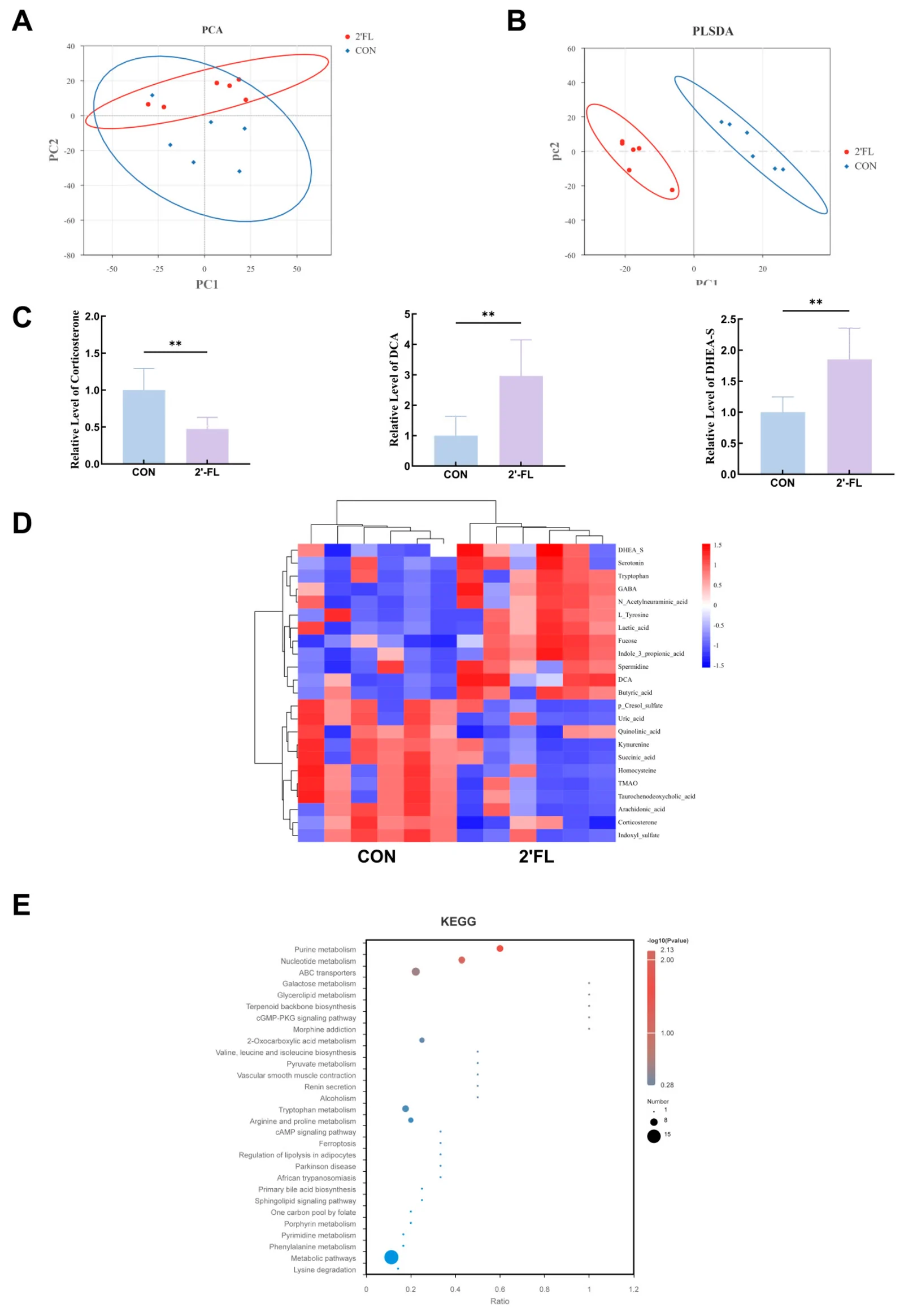
Serum metabolomic profiles and metabolic pathway analysis. (**A**) principal component analysis (PCA) scatter plot; (**B**) partial least squares-discriminant analysis (PLS-DA) score map; (**C**) normalized concentrations of dehydroepiandrosterone sulfate (DHEA-S), deoxycholic acid (DCA), and corticosterone; (**D**) heatmap illustrating altered metabolites; (**E**) KEGG pathway analysis for varied metabolites. Dots represent individual biological replicates.Data are presented as mean ± SD for quantitative panels. Statistical analysis was performed using an independent-samples *t* test or Mann–Whitney U test, as appropriate. ** *p* < 0.01.

PCA showed clear separation of the metabolic profiles between the two groups, indicating that 2′-FL intervention induced marked metabolic changes (Figure 5A). The PLS-DA model further enhanced the discrimination between groups. As shown in Figure 5B, the model exhibited good predictive performance without evident overfitting (R^2^ = 0.94, Q^2^ = 0.72).

A heatmap was constructed to illustrate metabolites fulfilling these filtering standards across the two cohorts. The investigation revealed that metabolites possessing neuroactive or potential defensive characteristics, such as dehydroepiandrosterone sulfate (DHEA-S), deoxycholic acid (DCA), and butyric acid, were notably increased in the 2′-FL cohort. Conversely, stress-related or microbial-derived metabolites, including corticosterone, kynurenic acid, and indoxyl sulfate, were considerably diminished (Figure 5C,D).

KEGG pathway investigations indicated that the distinct metabolites were primarily enriched in purine biosynthesis, nucleotide pathways, and ABC transporter-related pathways, suggesting that 2′-FL intervention may influence energy homeostasis and microbial-host metabolic interactions. Importantly, pathways associated with the gut–brain axis, such as tryptophan metabolism and primary bile acid biosynthesis, were also substantially enriched (Figure 5E).

In summary, the results indicate that neonatal 2′-FL intervention was associated with systemic metabolic remodeling. At the molecular level, this was characterized by increased levels of metabolites with potential neuroactive properties, including DHEA-S and DCA, together with reduced levels of stress-related metabolites such as corticosterone. At the pathway level, these changes were concentrated in core metabolic networks related to cellular energy status, signal transduction, and substrate transport. These metabolomic findings are consistent with the enhanced intestinal barrier function and reduced serum endotoxin-related markers observed above, collectively supporting the possibility that 2′-FL influences early-life physiology through the gut–brain axis [24,25].

### 3.6. 2′-FL Is Associated with Increased PSD95 Expression in the Hippocampal CA1 Region

**Figure 6:**
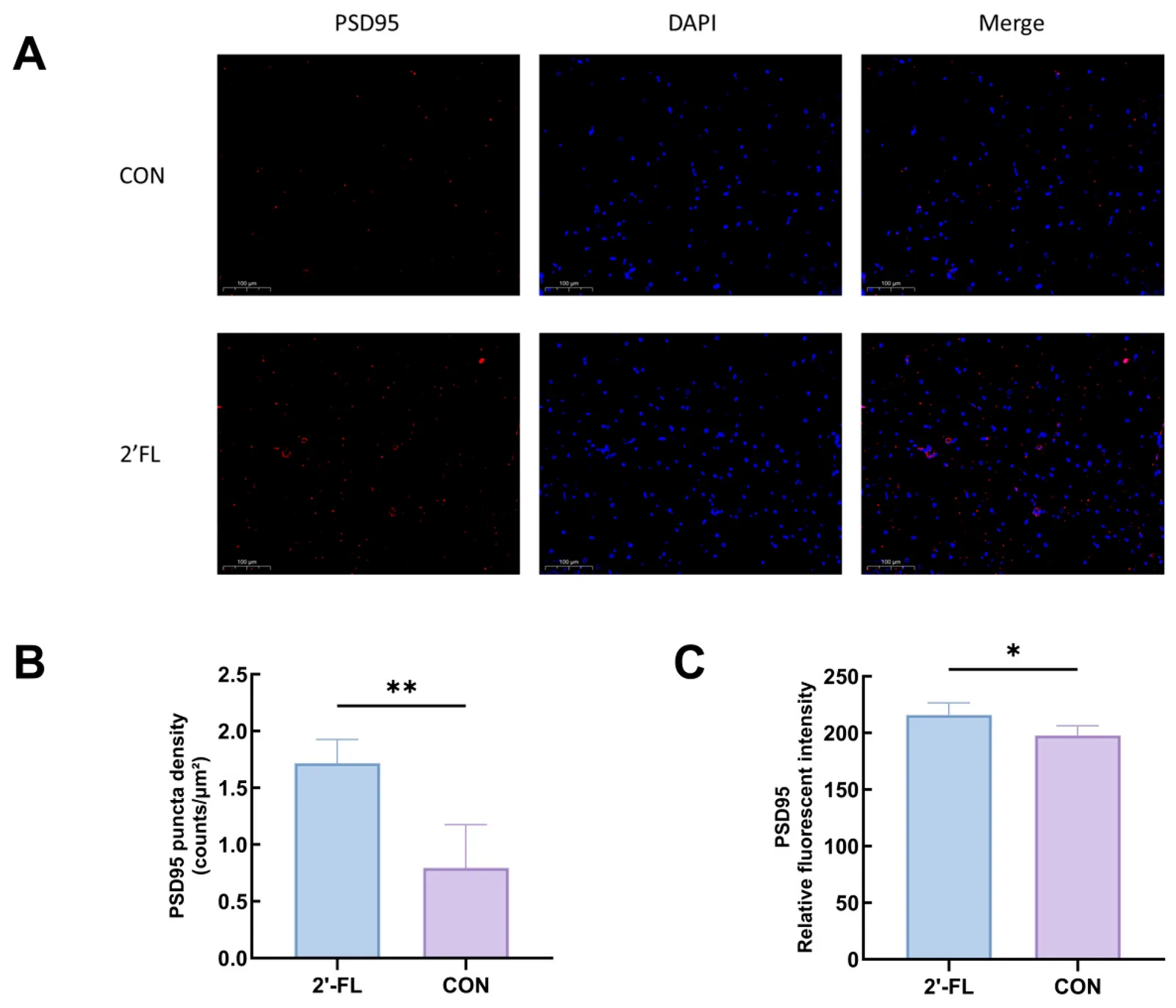
Hippocampal PSD95 expression in the CA1 region. (**A**) Representative immunofluorescence images of PSD95 (red) and DAPI nuclear staining (blue) in the hippocampal CA1 region; (**B**) quantification of PSD95-positive puncta density; (**C**) quantification of average PSD95 fluorescence intensity. Data are presented as mean ± SD. Statistical analysis was performed using an independent-samples *t* test or Mann–Whitney U test, as appropriate.* *p* < 0.05, ** *p* < 0.01.

To explore the potential effects of 2′-FL on hippocampal synaptic plasticity, immunofluorescence staining and quantitative analysis of PSD95, an excitatory postsynaptic marker, were performed in the stratum radiatum beneath the pyramidal cell layer of the hippocampal CA1 region. As a result, the PSD95 fluorescence signal in the hippocampal CA1 region of the 2′-FL group was stronger and displayed a denser punctate distribution (Figure 6A). Quantitative analysis further confirmed this difference: 2′-FL intervention significantly increased the average fluorescence intensity of PSD95 in the hippocampal CA1 region (*p* < 0.05). In parallel, the density of PSD95-positive puncta per unit area was also significantly increased (*p* < 0.05) (Figure 6B,C).

The consistent changes in these two indices suggest that neonatal 2′-FL supplementation was linked to enhanced levels of the excitatory postsynaptic marker PSD95 and may reflect enhanced synaptic structural plasticity in the hippocampus, a brain region closely involved in learning, memory, and emotional regulation [26].

### 3.7. Western Blot Validation of Hippocampal PSD95, BDNF, and Synaptophysin Expression

**Figure 7:**
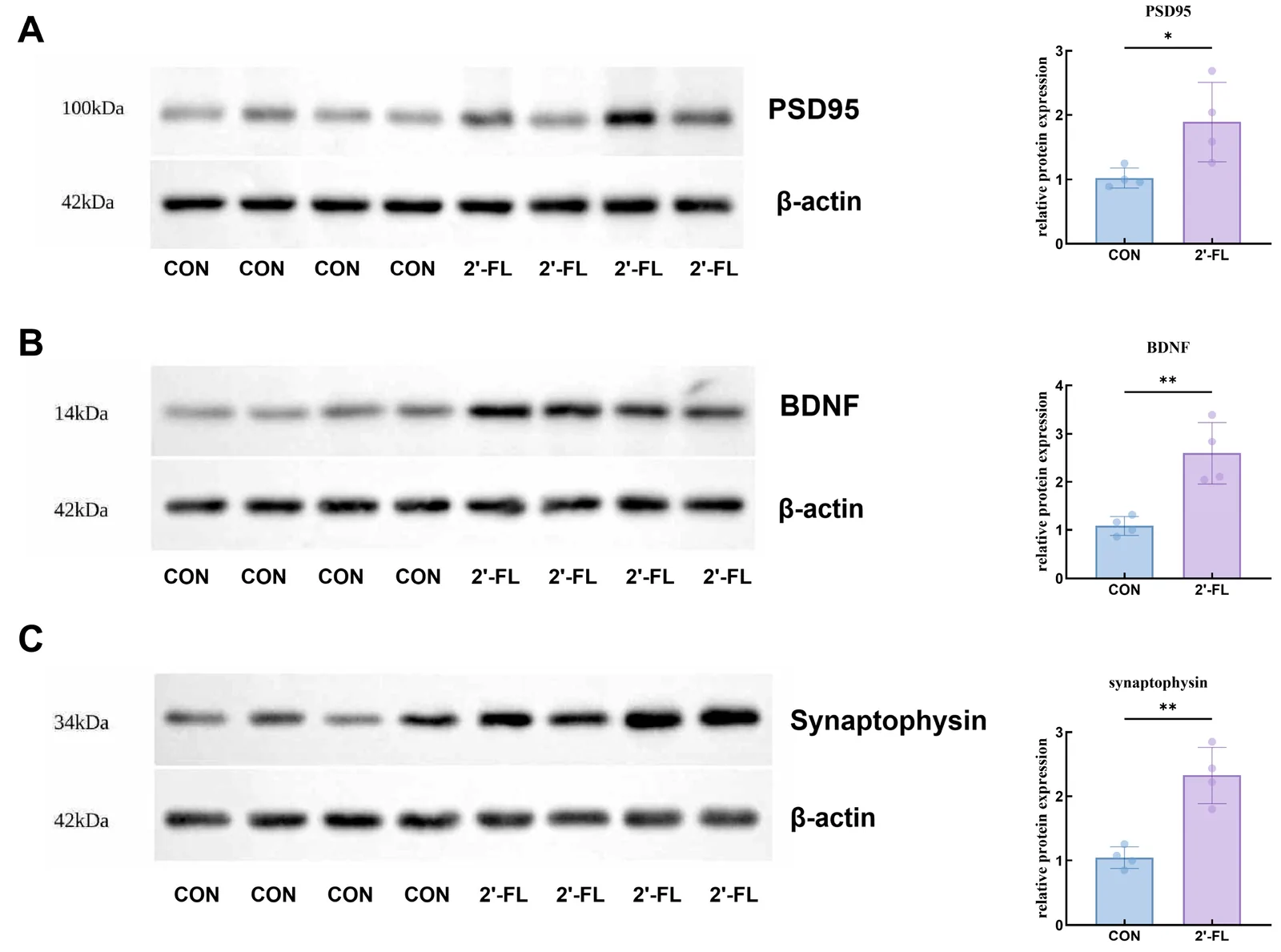
Western blot validation of hippocampal synaptic plasticity-related proteins. (**A**–**C**) Representative Western blot bands and quantitative analysis of postsynaptic density protein 95 (PSD95) (**A**), brain-derived neurotrophic factor (BDNF) (**B**), and synaptophysin (**C**), normalized to beta-actin. Data are presented as mean ± SD.Dots represent individual biological replicates. Statistical analysis was performed using an independent-samples *t* test or Mann–Whitney U test, as appropriate.* *p* < 0.05, ** *p* < 0.01.

To further evaluate whether the hippocampal changes observed by immunofluorescence were accompanied by broader synaptic plasticity-related protein alterations, Western blot analysis was performed for PSD95, BDNF, and Synaptophysin. The band patterns showed an overall upward trend in these proteins in the 2′-FL group compared with the CON group after normalization to beta-actin (Figure 7). PSD95 is closely related to postsynaptic structural organization, BDNF supports activity-dependent neuronal and synaptic remodeling, and Synaptophysin reflects presynaptic vesicle abundance and terminal integrity. Together with the enhanced PSD95 immunofluorescence in the hippocampal CA1 region and the improved exploratory behavior observed in the open field test, these Western blot results indicate that neonatal 2′-FL supplementation was associated with a coordinated shift toward enhanced hippocampal synaptic plasticity-related molecular profiles.

### 3.8. Spearman Correlation Analysis of the Microbiota-Metabolite-Brain Integrated Network

**Figure 8:**
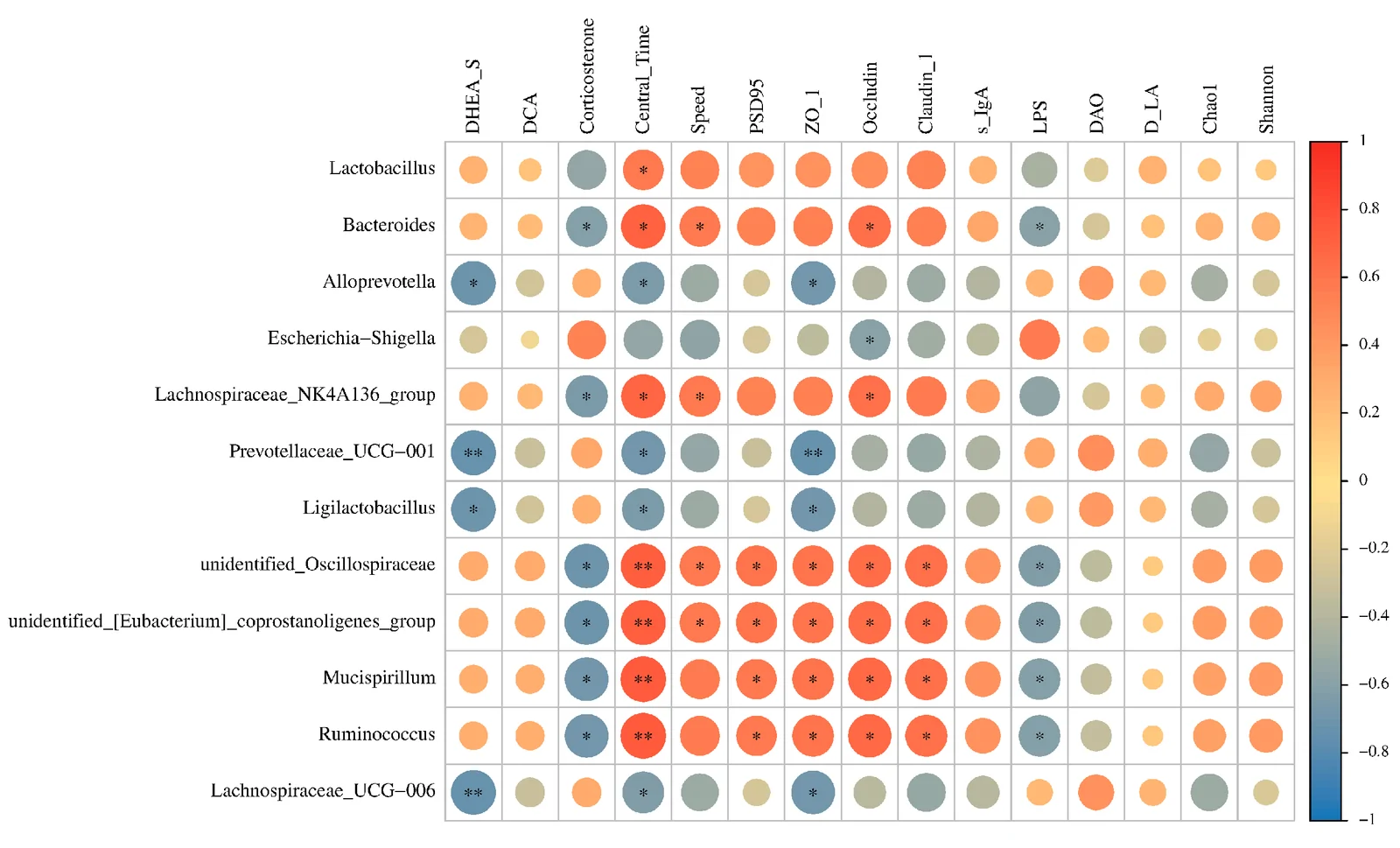
Spearman correlation analysis across key biological parameters. Spearman rank correlation analysis was used. Color intensity indicates Spearman correlation coefficients, and statistical significance is indicated in the correlation matrix.* *p* < 0.05, ** *p* < 0.01.

To further characterize the associations within the gut–brain axis, Spearman correlation analysis was performed between 10 differentially enriched bacterial taxa and 15 key indicators (Figure 8). The results showed that taxa significantly enriched by 2′-FL, including *Lactobacillus*, Lachnospiraceae_NK4A136_group, and *Bacteroides*, were positively correlated with metabolites of potential neurobiological relevance, including DHEA-S and DCA, as well as behavioral indicators including movement speed and central exploration time, and hippocampal PSD95 expression (*p* < 0.05). Meanwhile, these species exhibited a favorable association with the transcription levels of intestinal barrier genes, such as ZO-1, Occludin, and Claudin-1, as well as with s-IgA, but negatively correlated with markers of intestinal barrier damage and stress-related signals, including LPS, DAO, D-LA, and corticosterone.

Overall, the multidimensional correlation matrix supports the central hypothesis of this study, namely that 2′-FL-driven gut microbiota remodeling is closely associated with changes in systemic metabolism, intestinal homeostasis, and hippocampal synaptic plasticity-related markers during early life.

## 4. Discussion

Human milk oligosaccharides have attracted increasing attention as early-life nutritional factors that shape intestinal ecology and support infant development. Previous reviews and clinical observations have emphasized associations between human milk oligosaccharides, infant gut microbiota maturation, and neurodevelopmental outcomes [1,2,27]. Experimental studies have further shown that 2′-FL can promote beneficial microbial profiles, improve epithelial barrier-related functions, and modulate host physiological responses in cell, animal, and human-associated models [3,4,5,6,7,8,9,10]. Within this research landscape, the present study extends prior work by examining whether neonatal 2′-FL supplementation links microbial remodeling with systemic metabolism, intestinal barrier status, hippocampal synaptic plasticity-related proteins, and exploratory behavior in one integrated rat model.

A substantial body of work has already established the prebiotic properties of 2′-FL. In adults and in vitro fermentation models, 2′-FL or related human milk oligosaccharides altered microbial community structure and supported the growth of taxa with potential health relevance [4,7,8]. Other studies have shown that 2′-FL can enhance intestinal epithelial adhesion or barrier-associated functions, supporting the view that its biological effects are not limited to microbial composition alone [10,14]. Our findings are consistent with these observations, because 2′-FL supplementation was associated with increased abundance of *Lactobacillus*, *Bacteroides*, and *Lachnospiraceae_NK4A136_group*, together with reduced serum DAO, D-LA, and LPS. These results suggest that neonatal 2′-FL may support a gut environment characterized by both microbial restructuring and improved barrier-related status.

Compared with earlier studies that primarily focused on intestinal outcomes, increasing evidence indicates that 2′-FL may also influence neurodevelopmental and behavioral endpoints. Rodent studies have reported that 2′-FL supplementation during early life improved memory-related performance and enhanced long-term potentiation, with evidence implicating gut–brain communication through the vagus nerve [28,29]. A young pig study also linked 2′-FL and *Bifidobacterium longum* subsp. *infantis* supplementation with cognitive and structural brain development outcomes [15]. More recently, 2′-FL supplementation was reported to improve neurodevelopmental disorder-like phenotypes in offspring mice exposed to maternal immune activation [16]. Against this background, our study adds evidence from neonatal SD rats that 2′-FL supplementation is associated with greater exploratory activity and hippocampal synaptic plasticity-related protein expression.

The behavioral phenotype observed in this study was supported by convergent hippocampal molecular findings. The 2′-FL group showed increased PSD95 immunofluorescence in the CA1 region and higher hippocampal expression of PSD95, BDNF, and Synaptophysin. PSD95 is a core postsynaptic scaffold protein involved in excitatory synaptic organization, while BDNF contributes to activity-dependent neural circuit development and synaptic remodeling [30,31]. The parallel increase in postsynaptic, neurotrophic, and presynaptic markers strengthens the interpretation that neonatal 2′-FL supplementation was associated with enhanced hippocampal synaptic plasticity-related status.

The present results also align with the broader microbiota-gut–brain axis literature. Contemporary models propose that gut microorganisms communicate with the host through neural, endocrine, immune, metabolic, and barrier-related routes [1,11,32]. The neonatal period is especially relevant because microbial colonization, barrier maturation, metabolic programming, and brain circuit refinement occur during overlapping developmental windows. In this context, the reduced serum DAO, D-LA, and LPS levels after 2′-FL intervention indicate improved intestinal barrier-related status and lower exposure to translocation-associated inflammatory signals. Barrier-mediated communication has become an important focus in gut–brain research, as intestinal and brain-associated barriers regulate the access of peripheral signals to neurodevelopmentally relevant tissues [33].

A key contribution of this study is the inclusion of untargeted serum metabolomics alongside microbiota and hippocampal endpoints. Previous metabolic fate studies suggest that 2′-FL can directly influence gut microbial activity without necessarily accumulating in brain tissue [34]. This observation supports the interpretation that neurobehavioral effects of 2′-FL may arise through microbial and host-metabolic pathways rather than direct cerebral exposure. In our study, DHEA-S and DCA were increased, whereas corticosterone was decreased. Gut-derived metabolites have been shown to modulate brain activity and anxiety-related behavior in experimental models [35]. Therefore, the altered serum metabolic profile provides a plausible systemic link between intestinal microbial remodeling and hippocampal plasticity-related changes.

The endocrine-related findings are also biologically relevant. In rodents, corticosterone is a major stress-related glucocorticoid, and excessive hypothalamic–pituitary–adrenal axis activation can influence anxiety-related behavior and neurodevelopmental processes [36]. DHEA-S has been discussed as a neuroactive steroid with potential anti-glucocorticoid and stress-buffering properties [37]. The combination of lower corticosterone and higher DHEA-S in the 2′-FL group may therefore indicate a shift toward a more favorable stress-related endocrine profile. This interpretation is consistent with the behavioral changes observed in the open field test, although direct stress-challenge experiments would be needed to confirm this mechanism.

The increase in DCA further suggests that bile acid-related host-microbe co-metabolism may be involved. Bile acids are now recognized as signaling molecules that regulate metabolism, inflammation, epithelial function, and microbial ecology through reciprocal host-microbiota interactions [38]. The concurrent increase in DCA and enrichment of *Lactobacillus intestinalis* suggests that 2′-FL may have shifted bile acid-related metabolic output by reshaping the intestinal microbial ecosystem. Previous work has also linked DCA with microbiota-associated inflammatory signaling in gastrointestinal disease models [39]. In the present neonatal model, these findings provide a mechanistic lead connecting microbial remodeling, barrier-related status, and circulating metabolic signals.

Pathway enrichment analysis provided additional context for interpreting the metabolomic data. Altered metabolites were enriched in pathways related to purine metabolism, nucleotide metabolism, transport processes, and intracellular signaling, including cAMP and cGMP-PKG-related pathways. These pathways are closely connected to cellular energy balance, signal transduction, and synaptic adaptation. Although enrichment analysis cannot establish causality by itself, it identifies metabolic routes that may help explain how neonatal 2′-FL supplementation coordinates intestinal, systemic, and hippocampal responses.

Taken together, this study advances the current understanding of 2′-FL by moving beyond a single-outcome description of its prebiotic activity. The findings support a working model in which neonatal 2′-FL reshapes gut microbial ecology, improves intestinal barrier-related status, and alters circulating metabolites, including neuroactive steroids, bile acid-related metabolites, and stress-associated endocrine signals. These peripheral changes may converge on hippocampal synaptic plasticity-related pathways and contribute to altered exploratory behavior. Future studies using fecal microbiota transfer, targeted metabolomics, shotgun metagenomics, pathway perturbation, and additional behavioral paradigms will help clarify causality and refine the translational relevance of these findings for infant nutrition research.

## 5. Conclusions

This study demonstrates that supplementation with 2′-fucosyllactose during early life enhances locomotor activity and exploratory behavior in neonatal rats and is associated with increased expression of hippocampal synaptic plasticity-related markers, including PSD95, BDNF, and synaptophysin. The underlying mechanism may involve regulation along the microbiota-gut–brain axis: 2′-FL reshaped the gut microbiota and was accompanied by remodeling of the serum metabolomic profile, characterized by increased levels of the neuroactive steroid DHEA-S and the secondary bile acid DCA, together with reduced levels of stress-related corticosterone. These coordinated intestinal, metabolic, and neurobiological changes may provide supportive signals for hippocampal synaptic development and plasticity, ultimately contributing to altered behavioral performance. This study provides experimental evidence and mechanistic clues supporting the potential application of 2′-FL in infant nutrition for early neurodevelopmental support.

## Data Availability

The original contributions presented in this study are included in the article/Supplementary Materials. Further inquiries can be directed to the corresponding authors.

## Supplementary Materials

The following supporting information can be downloaded at: https://www.mdpi.com/article/10.3390/foods15173151/s1, Table S1: Specific Primer Sequences for RT-qPCR Analysis.
