# Supplementary material for: Neonatal 2′-Fucosyllactose Supplementation Modulates Exploratory Behavior and Hippocampal Synaptic Plasticity in Rats Through Gut Microbiota and Metabolic Remodeling
Source: Foods. 2026 Sep 5;15(17):3151. doi: 10.3390/foods15173151 (PMC13565205; doi:10.3390/foods15173151)
Supplement: Supplementary file 1 [file foods-15-03151-s001.zip › foods-4501361-supplementary.pdf]

Table S1. Specific Primer Sequences for RT-qPCR Analysis.

Genes	Forward primer (5′→3′)	Reverse primer (5′→3′)
β-actin	GGCTGTATTCCCCTCCATCG	CCAGTTGGTAACAATGCCATGT
ZO-1	GGGGCCTACACTGATCAAGA	TGGAGATGAGGCTTCTGCTT
Occludin	GGAATGTCCAGGACAAGAAG	GCCGTACATAGATCCAGAAG
Claudin-1	AAAGCACCGGGCAGATACAG	CCCAGCAGGATGCCAATTAC
